# Development and validation of an interpretable machine learning model for the early diagnosis of invasive pulmonary aspergillosis in patients with severe fever with thrombocytopenia syndrome: a retrospective cohort study

**DOI:** 10.3389/fcimb.2026.1857200

**Published:** 2026-07-30

**Authors:** Shuwen Ding, Ruize Ma, Wan Peng, Yanli Xu, Ling Lin, Zhihai Chen

**Affiliations:** 1 National Key Laboratory of Intelligent Tracking and Forecasting for Infectious Diseases, Beijing Ditan Hospital, Capital Medical University, Beijing, China; 2 Department of Infectious Diseases, Yantai City Hospital for Infectious Disease, Yantai, China

**Keywords:** early diagnosis, invasive pulmonary aspergillosis, machine learning, predictive models, severe fever with thrombocytopenia syndrome

## Abstract

**Objectives:**

Invasive pulmonary aspergillosis (IPA) is a common complication in patients with severe fever with thrombocytopenia syndrome (SFTS); however, its diagnosis remains challenging due to non-specific clinical manifestations and limited diagnostic tools. This study aimed to develop and validate a machine learning (ML)-based early diagnostic model to identify IPA in SFTS patients.

**Methods:**

A total of 374 SFTS patients with suspected IPA were retrospectively enrolled at Qishan Hospital between March 2022 and June 2025. Both the Least Absolute Shrinkage and Selection Operator (LASSO) and the Boruta algorithms were employed for feature selection. Seven ML algorithms were subsequently developed and evaluated using multiple evaluation metrics to compare their predictive performance. Optimal cutoff values were determined by the Youden index. The AUCs of the models were compared using the DeLong test. Feature contributions in the optimal model were interpreted using SHapley Additive exPlanations (SHAP).

**Results:**

Among the seven algorithms, the Random Forest (RF) model demonstrated the best predictive performance. The final model incorporated five key features: sex, serum potassium, international normalized ratio (INR), galactomannan (GM) test positivity, and SFTSV RNA load. The RF model achieved an area under the receiver operating characteristic curve (AUC) of 0.871 (95% CI: 0.799-0.933), with a specificity of 0.829 (95% CI: 0.735-0.912), a sensitivity of 0.854 (95% CI: 0.738–0.953), and an F1 score of 0.795. Calibration was acceptable, with a Brier score of 0.140 (95% CI: 0.105 - 0.179), and clinical utility was confirmed through decision curve analysis (DCA).

**Conclusions:**

Our ML model provides a non-invasive and cost-effective approach for the early identification of SFTS-associated IPA. It may serve as a valuable adjunct to existing diagnostic strategies and support timely diagnostic evaluation in high-risk patients. Further external validation is warranted before clinical implementation.

## 1 Introduction

Severe Fever with Thrombocytopenia Syndrome (SFTS) is a tick-borne zoonotic disease caused by the Severe Fever with Thrombocytopenia Syndrome Virus (SFTSV). Since its first identification in rural areas of Hubei and Henan provinces in Central China in 2009, SFTS cases have been reported in multiple countries across East and Southeast Asia (Ohno et al., 2025). Due to its high case fatality rate and the growing geographic spread (Casel et al., 2021; Seo et al., 2021), the World Health Organization (WHO) and the National Institute of Allergy and Infectious Diseases (NIAID) of the United States designated SFTS as a priority pathogen (Kim et al., 2024).

Patients with SFTS exhibit a high prevalence of bacterial and fungal co-infections, ranging from 27.4% to 45.0% (Song et al., 2023a; Hu et al., n.d). Among these, invasive pulmonary aspergillosis (IPA)—conventionally observed in immunocompromised hosts—has emerged as a particularly life-threatening complication in critically ill SFTS patients (Zhao et al., 2025b; Castro-Fuentes et al., 2026). Recent studies have reported a high incidence of IPA in SFTS patients, ranging from 21.2% to 56%, with associated mortality rates ranging from 25.42% to 37.9% (Song et al., 2023b; Wang et al., 2024; Xu et al., n.d).

However, early identification of SFTS-associated IPA (SAPA) remains challenging due to the lack of specific clinical manifestations and the limitations of currently available diagnostic methods. Conventional fungal cultures of respiratory specimens are time-consuming and often lack sufficient sensitivity. Although serum galactomannan (GM) is one of the most widely used biomarkers for IPA diagnosis, its diagnostic performance is suboptimal in non-neutropenic patients like SFTS patients (Lu et al., 2023; Niu et al., 2024). While galactomannan detection in bronchoalveolar lavage fluid (BALF) generally demonstrates higher sensitivity than in plasma, its overall diagnostic performance remains imperfect and requires invasive sampling procedures (Sun et al., n.d). Consequently, the diagnosis of SAPA is frequently delayed, with a reported median time to diagnosis of approximately 8 days (Seong et al., 2021). Such delays may postpone the initiation of antifungal therapy and adversely affect patient outcomes, highlighting the need for more effective approaches for the early identification of SFTS-associated IPA.

Therefore, this retrospective study aimed to develop and validate an integrated diagnostic model for the early identification of patients at high risk of SFTS-associated IPA. By incorporating multiple clinical and laboratory variables, the model may serve as an adjunctive tool to support early diagnostic evaluation and clinical risk stratification of patients with SFTS-associated IPA in real-world settings.

## 2 Methods

### 2.1 Patients and study design

Patient data were collected from individuals diagnosed with SFTS at Qishan Hospital in Yantai between March 2022 and June 2025. Inclusion criteria included:(a) a confirmed diagnosis of SFTS based on the detection of SFTSV by reverse transcription polymerase chain reaction (RT-PCR) (NHCotPsRo, 2023); and (b) age ≥ 18 years. Exclusion criteria were patients who (a) had pre-existing immunodeficiency disorders; (b) had autoimmune diseases or malignancies; (c) had other concurrent invasive fungal infections; (d) were missing critical laboratory results or clinical documentation; (e) were pregnant; (f) died within 24h of admission; (g) were receiving systemic antifungals >48h pre-admission; or (h) had pre-existing unresolved IPA before SFTSV infection.

The enrolled cohort was categorized into IPA and non-IPA groups based on established diagnostic guidelines. In the absence of universally accepted diagnostic criteria for SFTS-associated IPA, IPA was diagnosed according to the guidelines of the European Organization for Research and Treatment of Cancer and the Mycoses Study Group Education and Research Consortium (EORTC/MSGERC) updated in 2020 (Donnelly et al., 2019), consistent with the methodology adopted in previous studies of SFTS-related IPA (Song et al., 2023b; Yan et al., 2025; Xu et al., n.d). Patients meeting the modified EORTC/MSGERC criteria for probable IPA were assigned to the IPA group, whereas all remaining patients were assigned to the non-IPA group. No proven IPA cases were identified in the study cohort.

### 2.2 Data collection

All the information was collected from the electronic hospital records system. The following data from each patient were reviewed: (a) Patients’ background, including sex, age, underlying disease, onset to admission interval; (b) Clinical manifestations, comprising fever, fatigue, cough, expectoration, dyspnea, central nervous system symptoms, and lymphadenopathy; (c) Laboratory testing, including complete blood count, serum chemistry, coagulation system tests, serum 1,3-β-D-glucan test (G test), galactomannan test (GM test), and viral load.

Serum G and GM tests were performed according to routine clinical practice at the discretion of the treating physician when fungal infection was clinically considered. Baseline clinical and laboratory parameters were collected within the first 48 hours of admission. To preserve the temporal relationship between predictors and outcome, only variables obtained at least 72 hours before the diagnosis of IPA were included in the analysis.

CNS symptoms were defined as unexplained altered mental status (including impaired consciousness, lethargy, or personality changes) persisting for ≥24 h, or generalized/focal seizures not attributable to a preexisting seizure disorder (Shan et al., 2024; Wu et al., 2025).

### 2.3 Data preprocessing

The dataset was divided into a training set (70%) and a validation set (30%) using stratified sampling based on the IPA diagnosis. All candidate predictors exhibited low levels of missingness (<5% in both the training and validation sets). To reduce bias and improve statistical power, missing values were imputed via multiple imputation using the MICE package in R. Specifically, five imputed datasets were generated (m=5), with a random seed of 123 set for reproducibility.

### 2.4 Variable selection and model development

Feature selection was performed using the Least Absolute Shrinkage and Selection Operator (LASSO) regression and the Boruta algorithm. LASSO regression with 10-fold cross-validation was first applied to determine the optimal penalty parameter, shrinking the coefficients of less important predictor variables to zero and retaining variables with non-zero coefficients, thereby reducing redundant predictors (Zeng et al., n.d). Boruta, a random forest–based feature selection algorithm, was subsequently used to identify variables whose importance consistently exceeded that of randomly generated shadow features (Wang et al., n.d). As the two methods identify informative predictors using different selection principles, only variables selected by both algorithms were retained for final model construction to improve feature-selection stability while maintaining a parsimonious model.

In this study, seven common binary classifiers—Logistic Regression (LR), Decision Tree (DT), Support Vector Machine (SVM), Random Forest (RF), Light Gradient Boosting Machine (LightGBM), Extreme Gradient Boosting (XGBoost), and Artificial Neural Network (ANN)—were used to construct the prediction models.

Hyperparameter tuning was performed on the training set using grid search with 5-fold cross-validation. The performance of the final optimal model was then evaluated on the validation set.

### 2.5 Model validation

The performance of the prediction models was quantified through accuracy, precision, sensitivity, specificity, F1 score, Kappa coefficient, and Youden index, with the classification outcomes visualized through a confusion matrix. The optimal classification threshold for each model was determined by the maximum Youden index.

Receiver operating characteristic (ROC) curves, calibration plots, and decision-curve analysis (DCA) were used to assess discrimination, calibration, and net clinical utility (Shipe et al., 2019). Uncertainty for the area under the curve (AUC) and Brier score was quantified by bootstrap resampling of the validation set (1,000 iterations). Differences in discriminative performance between competing models were assessed using DeLong’s test. Bootstrap resampling (5,000 iterations) was performed to estimate 95% confidence intervals for differences in ΔAUC between models.

### 2.6 Model interpretation

SHAP (SHapley Additive exPlanations) values were employed to quantify the contribution of each feature to the model’s output. Features with higher absolute SHAP values were deemed to exert a greater effect on the model predictions. This approach enhanced model interpretability and facilitated clinical understanding of model decisions.

### 2.7 Statistical analysis

All statistical analyses were performed using R (v4.5.1; R Foundation for Statistical Computing, Vienna, Austria). The normality of continuous variables was assessed using the Shapiro–Wilk test. Accordingly, normally distributed data were expressed as mean ± standard deviation (SD) and compared via the Student’s t-test, while non-normally distributed data were presented as median with interquartile range (IQR) and analyzed with the Mann–Whitney U test. Categorical variables were presented as counts and percentages, with group differences compared using the χ^2^ or Fisher’s exact test, as appropriate. A two-sided p-value < 0.05 was regarded as statistically significant.

### 2.8 Sensitivity analyses

To evaluate the potential risk of target leakage arising from the inclusion of GM test positivity, which forms part of the diagnostic criterion for IPA, a sensitivity analysis was conducted. All seven machine learning models were retrained after excluding GM test positivity from the predictor set. The restricted models were evaluated using the same model-development framework as in the primary analysis. Their performance was subsequently compared with that of the original models to assess the influence of GM positivity on predictive performance.

## 3 Results

### 3.1 Baseline characteristics

Among the 374 patients with confirmed SFTS, 139 were classified as IPA, and 235 as non-IPA. Baseline characteristics were compared between the two groups (**Table 1**).

**Table 1:** Baseline characteristics in this study.

Variables	Overall (n=374)	Non-IPA (n=235)	IPA (n=139)	*P*-value
Sex (Female), n (%)	208 (55.61)	125 (53.19)	83 (59.71)	0.263
Age (years)	67.00 [60.00, 74.00]	67.00 [59.00, 73.50]	70.00 [61.50, 75.00]	0.03
Onset to admission interval (days)	5.00 [4.00, 7.00]	5.00 [4.00, 7.00]	5.00 [4.00, 7.00]	0.715
Diabetes (%)	67 (17.91)	37 (15.74)	30 (21.58)	0.199
Hypertension (%)	91 (24.33)	54 (22.98)	37 (26.62)	0.504
Symptoms and signs				
Fever	344 (91.98)	219 (93.19)	125 (89.93)	0.355
Fatigue	282 (75.40)	174 (74.04)	108 (77.70)	0.504
Cough	62 (16.58)	37 (15.74)	25 (17.99)	0.675
Expectoration	49 (13.10)	28 (11.91)	21 (15.11)	0.468
Dyspnea	25 (6.68)	11 (4.68)	14 (10.07)	0.071
CNS symptoms	156 (41.71)	78 (33.19)	78 (56.12)	<0.001
Lymphadenopathy	97 (25.94)	55 (23.40)	42 (30.22)	0.183
Laboratory test				
NEU (10^9^/L)	1.33 [0.87, 2.11]	1.29 [0.87, 2.04]	1.42 [0.91, 2.32]	0.228
LY (10^9^/L)	0.44 [0.31, 0.66]	0.47 [0.32, 0.73]	0.39 [0.30, 0.56]	0.01
MO (10^9^/L)	0.12 [0.07, 0.29]	0.13 [0.08, 0.26]	0.11 [0.06, 0.36]	0.714
EO (10^9^/L)	0.00 [0.00, 0.01]	0.00 [0.00, 0.01]	0.00 [0.00, 0.01]	0.258
BA (10^9^/L)	0.00 [0.00, 0.01]	0.00 [0.00, 0.01]	0.00 [0.00, 0.01]	0.102
RBC (10^12^/L)	4.64 [4.30, 4.99]	4.62 [4.28, 4.97]	4.67 [4.32, 5.03]	0.59
HGB (g/L)	141.00 [130.00, 153.00]	141.00 [129.00, 152.00]	142.00 [132.00, 156.00]	0.337
PLT (10^9^/L)	61.50 [48.00, 81.00]	66.00 [51.00, 88.00]	57.00 [41.50, 71.00]	<0.001
MPV (fL)	10.30 [9.72, 11.00]	10.20 [9.70, 11.00]	10.30 [9.80, 11.10]	0.233
CRP (mg/L)	4.12 [1.50, 10.07]	3.13 [1.17, 8.86]	5.85 [2.41, 12.21]	<0.001
PCT (ng/ml)	0.19 [0.09, 0.48]	0.15 [0.08, 0.33]	0.30 [0.13, 0.66]	<0.001
CK-MB (U/L)	9.24 [5.00, 15.00]	9.00 [4.74, 14.00]	11.00 [6.16, 16.34]	0.007
UREA (mmol/L)	6.30 [4.68, 8.65]	5.89 [4.46, 7.81]	7.09 [4.98, 10.29]	0.001
CREA (umol/L)	68.00 [53.25, 85.00]	66.00 [53.00, 79.00]	74.80 [54.85, 97.50]	0.014
Na (mmol/L)	135.00 [131.93, 138.00]	135.00 [132.00, 138.00]	135.00 [131.55, 138.00]	0.807
K (mmol/L)	3.70 (0.48)	3.62 (0.45)	3.83 (0.50)	<0.001
INR	0.98 [0.93, 1.04]	0.96 [0.92, 1.02]	1.01 [0.96, 1.07]	<0.001
APTT (s)	49.90 [43.60, 57.58]	47.40 [42.20, 54.30]	54.50 [48.10, 64.95]	<0.001
TT (s)	22.95 [20.20, 27.60]	21.90 [19.65, 25.70]	25.70 [21.15, 32.70]	<0.001
FIB (g/L)	2.50 [2.08, 2.86]	2.54 [2.15, 2.90]	2.44 [2.02, 2.84]	0.049
ALT (U/L)	72.60 [42.78, 134.25]	67.90 [39.10, 118.90]	77.20 [50.35, 154.00]	0.01
AST (U/L)	149.00 [86.62, 305.45]	124.50 [68.10, 241.22]	201.20 [119.55, 463.65]	<0.001
TBIL (umol/L)	9.52 [7.60, 12.26]	9.44 [7.72, 11.95]	10.03 [7.52, 12.61]	0.538
DBIL (umol/L)	2.85 [2.23, 4.22]	2.72 [2.22, 3.86]	3.03 [2.33, 4.90]	0.028
ALB (g/L)	31.85 [29.10, 35.48]	32.40 [29.40, 35.80]	30.70 [28.40, 34.20]	0.002
GLOB (g/L)	27.00 [24.40, 29.40]	27.50 [25.10, 29.95]	26.40 [23.35, 28.55]	0.001
GGT (U/L)	28.00 [19.00, 54.00]	27.00 [18.00, 47.50]	28.00 [21.00, 60.50]	0.038
ALP (U/L)	60.90 [48.52, 79.25]	61.90 [49.50, 78.30]	59.30 [47.10, 80.10]	0.784
Positive G test	65 (17.38)	25 (10.64)	40 (28.78)	<0.001
Positive GM test	90 (24.06)	21 (8.94)	69 (49.64)	<0.001
SFTSV RNA (log_10_ TCID50/mL)	3.61 [2.63, 4.45]	3.04 [2.32, 3.97]	4.36 [3.65, 5.22]	<0.001

CNS, Central nervous system; NEU, Neutrophil; LY, Lymphocyte; MO, Monocyte; EO, Eosinophil; BA, Basophil; RBC, Red blood cell; HGB, Hemoglobin; PLT, Platelet; MPV, Mean platelet volume; CRP, C-reactive protein; PCT, Procalcitonin; CK-MB, Creatine kinase isoenzyme MB; CREA, Creatinine; Na, Sodium; K, Potassium; INR, International Normalized Ratio; APTT, Activated partial thromboplastin time; TT, Thrombin time; FIB, Fibrinogen; ALT, Alanine aminotransferase; AST, Aspartate aminotransferase; TBIL, Total Bilirubin; DBIL, Direct Bilirubin; ALB, Albumin; GLOB, Globulin; ALP, Alkaline Phosphatase; GGT, Gamma-glutamyl transferase; G test, 1,3-β-D-Glucan test; GM, Galactomannan.

Compared with the non-IPA group, patients with IPA exhibited significantly elevated levels of C-reactive protein (CRP), procalcitonin (PCT), serum potassium, aspartate aminotransferase (AST), and SFTSV RNA, as well as significantly prolonged coagulation parameters, whereas the PLT count was significantly lower (all *P* < 0.001).

### 3.2 Feature selection

**Figure 1:**
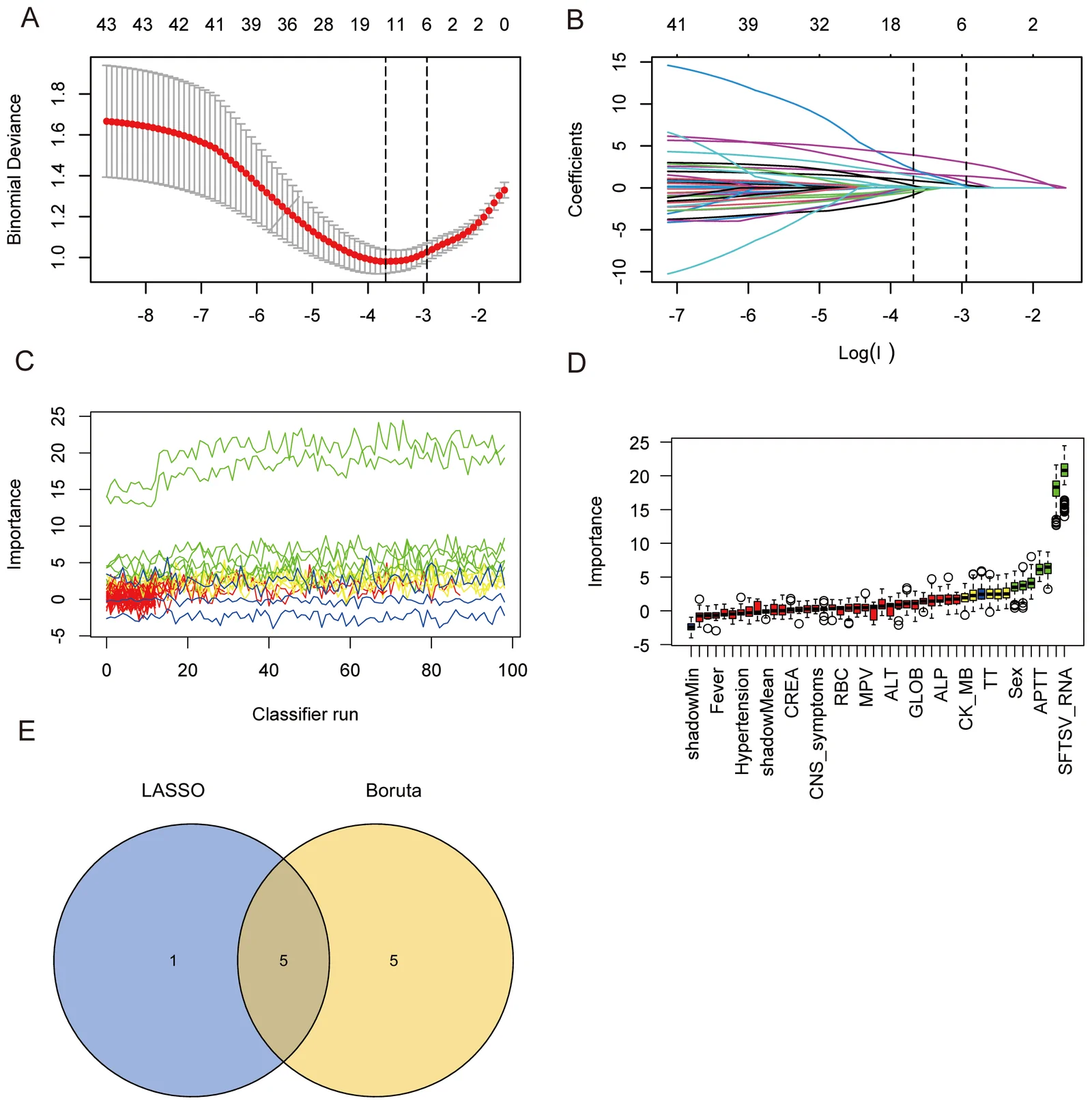
Variable selection process. **(A, B)** Feature selection using LASSO regression; **(C, D)** Feature identification via the Boruta algorithm; **(E)** Venn diagram showing the final five variables.

As shown in **Figure 1**, based on the λ_1se_ criterion, LASSO selected six variables with non-zero coefficients: sex, serum potassium, international normalized ratio (INR), gamma-glutamyl transferase (GGT), SFTSV RNA load, and GM test positivity. The Boruta algorithm identified ten key predictors: sex, serum potassium, monocyte count, INR, activated partial thromboplastin time (APTT), thrombin time (TT), AST, SFTSV RNA load, G test positivity, and GM test positivity. The intersection of these variables—sex, serum potassium, INR, GM test positivity, and SFTSV RNA load—was incorporated into the final model.

### 3.3 Model development and performance comparison

Seven predictive algorithms were developed to estimate the probability of IPA in patients with SFTS. Model hyperparameters were optimized via grid search and cross-validation, and the final tuned parameters for each model were summarized in **Table 2**.

**Table 2:** Optimal parameters of each model.

Model	Optimal parameters
Decision tree	‘ccp_alpha’: 0.0, ‘max_depth’: 3, ‘max_features’: None, ‘min_samples_split’: 20
Random forest	n_estimators = 400, max_features = 2
XGBoost	‘learning_rate’: 0.01, ‘max_depth’: 3, ‘n_estimators’: 200, ‘subsample’: 0.6
LightGBM	‘colsample_bytree’: 0.8, ‘learning_rate’: 0.1, ‘n_estimators’: 100, ‘num_leaves’: 31, ‘subsample’: 0.6
SVM	‘C’: 0.1, ‘degree’: 2, ‘gamma’: ‘scale’, ‘kernel’: ‘linear’
ANN	‘activation’: ‘tanh’, ‘hidden_layer_sizes’: (50, 50)

XGBoost, Extreme Gradient Boosting; LightGBM, Light Gradient Boosting Machine; SVM, Support Vector Machine; ANN, Artificial Neural Network.

**Figure 2:**
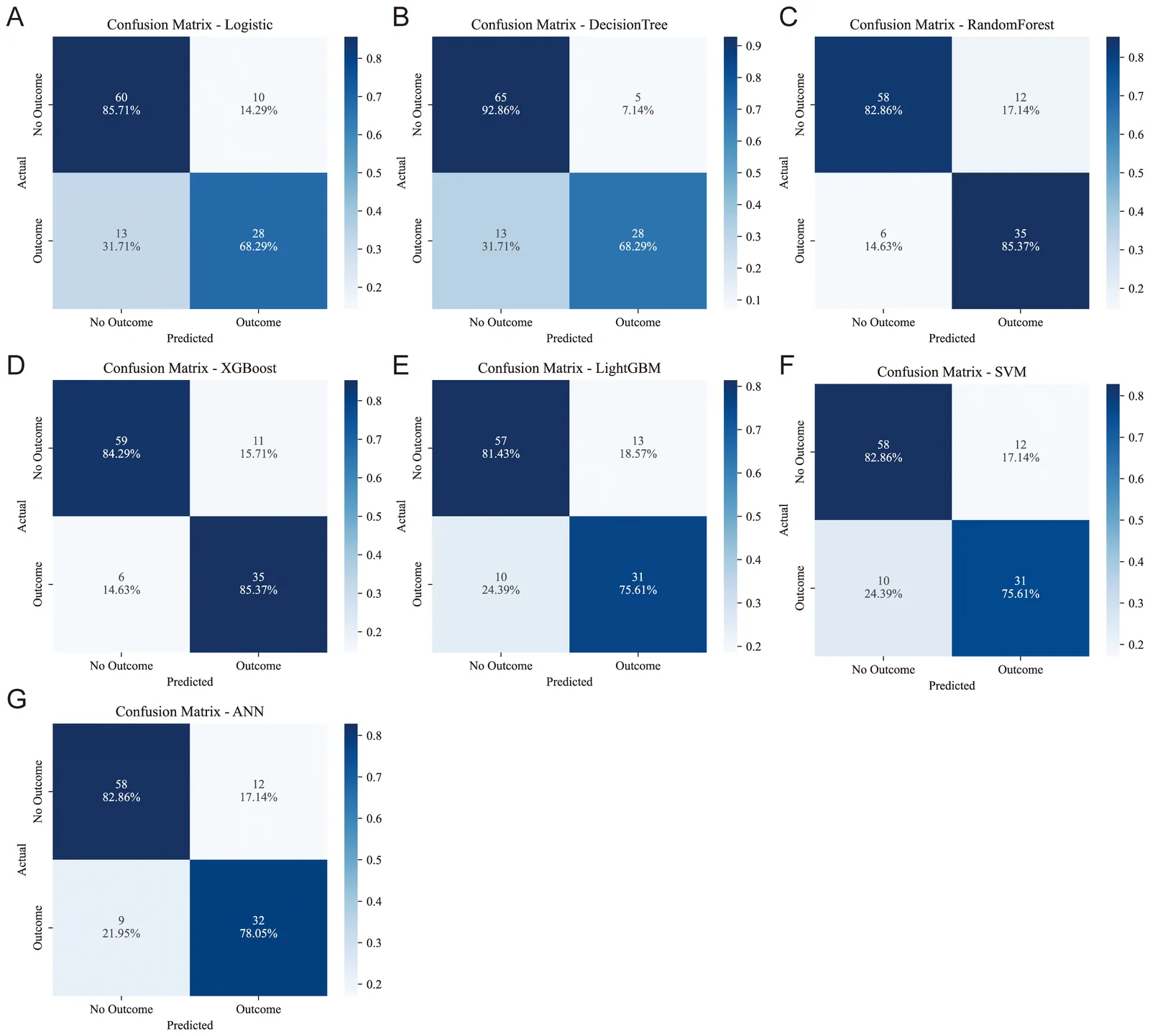
Confusion matrices of the seven machine learning models in the validation set. **(A)** Logistic regression; **(B)** Decision tree; **(C)** Random forest; **(D)** XGBoost; **(E)** LightGBM; **(F)** Support vector machine (SVM); **(G)** Artificial neural network (ANN). Actual outcomes are presented on the vertical axis, while predicted outcomes are on the horizontal axis.

The performance of the seven binary classifiers in the validation set was assessed via confusion matrices (**Figure 2**). Key metrics, including sensitivity, specificity, precision, negative predictive value, and accuracy, were then derived to compare their classification performance (Cabot and Ross, 2023).

**Figure 3:**
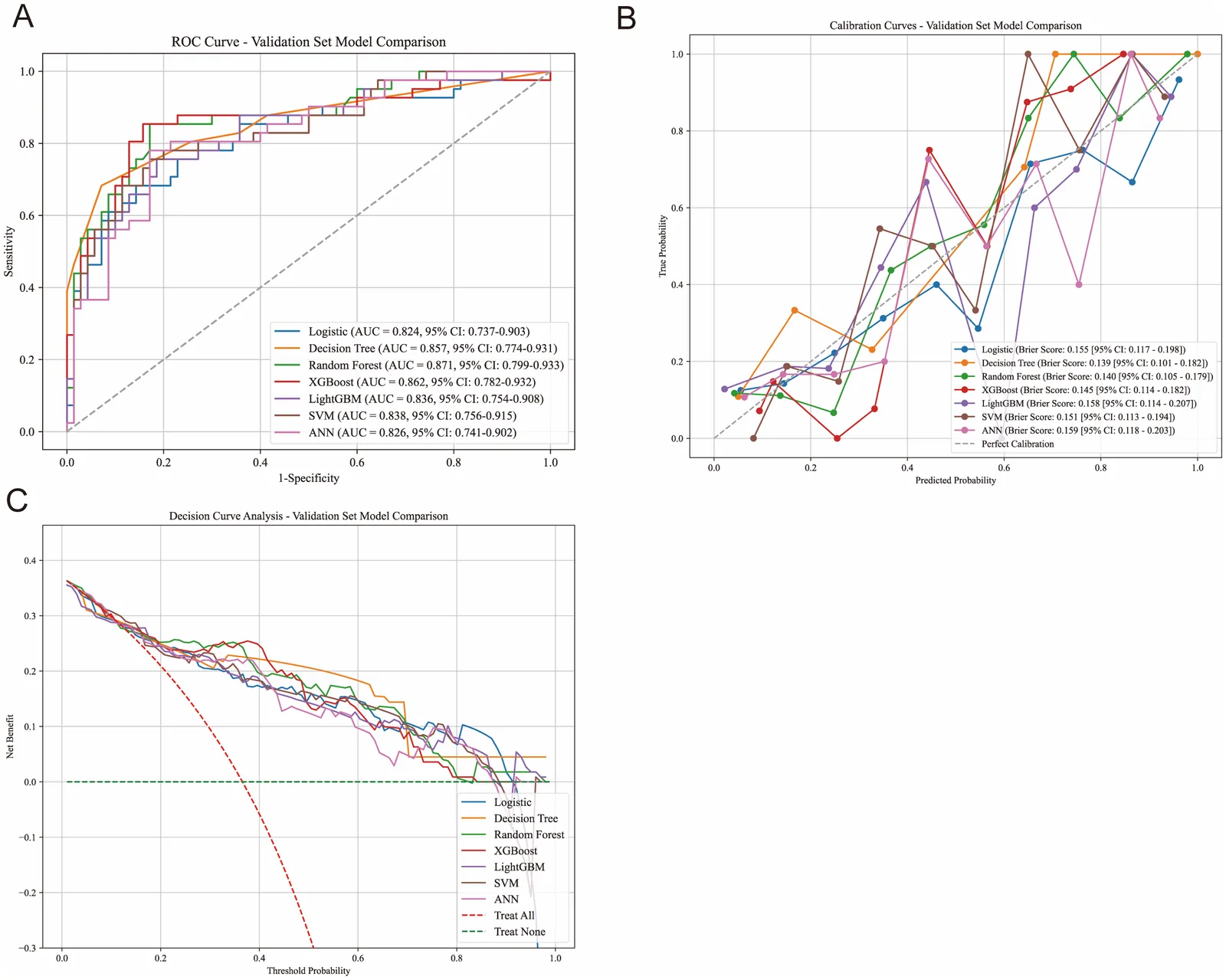
Performance comparison of the seven machine learning predictive models in the validation set. **(A)** Receiver operating characteristic (ROC) curves; **(B)** Calibration curves; **(C)** Decision curve analysis (DCA).

As presented in **Table 3**; **Figure 3A**, the RF model achieved the highest AUC (0.871, 95% CI: 0.799–0.933). Using the optimal cutoff probability of 0.3675 determined by the maximum Youden index (J = 0.682), the RF model achieved an accuracy of 0.838, a specificity of 0.829 (95% CI: 0.735-0.912), a sensitivity of 0.854 (95% CI: 0.738–0.953), and a precision of 0.745, reflecting favorable discriminative ability.

**Table 3:** Performance comparison of models on the validation set.

Model	AUC	95% CI Lower	95% CI Upper	Accuracy	Precision	Sensitivity	Specificity	F1 Score	Kappa	Youden’s J
Logistic	0.824	0.737	0.903	0.793	0.737	0.683	0.857	0.709	0.548	0.540
Decision Tree	0.857	0.774	0.931	0.838	0.848	0.683	0.929	0.757	0.637	0.611
Random Forest	0.871	0.799	0.933	0.838	0.745	0.854	0.829	0.795	0.662	0.682
XGBoost	0.862	0.782	0.932	0.847	0.761	0.854	0.843	0.805	0.679	0.697
LightGBM	0.836	0.754	0.908	0.793	0.705	0.756	0.814	0.729	0.562	0.570
SVM	0.838	0.756	0.915	0.802	0.721	0.756	0.829	0.738	0.579	0.585
ANN	0.826	0.741	0.902	0.811	0.727	0.780	0.829	0.753	0.600	0.609

XGBoost, Extreme Gradient Boosting; LightGBM, Light Gradient Boosting Machine; SVM, Support Vector Machine; ANN, Artificial Neural Network.

Pairwise DeLong tests were performed to compare model discrimination. Although the XGBoost model demonstrated a comparable AUC (0.862, 95% CI: 0.782–0.932) and a slightly higher F1 score (0.805), the difference in AUC between RF and XGBoost was not statistically significant (p = 0.536). Bootstrap resampling yielded consistent findings, with a mean ΔAUC of 0.009 (95% CI: -0.020-0.039). In contrast, RF demonstrated significantly better discrimination than Logistic Regression (p = 0.037), and bootstrap analysis showed a mean ΔAUC of 0.047 (95% CI: 0.005-0.094). Other models—including SVM, Decision Tree, and ANN—yielded acceptable levels of discrimination but were surpassed by the RF model. Given its highest AUC, favorable overall performance across multiple evaluation metrics, and good calibration and clinical utility (**Figures 3B, C**), RF was selected as the final model.

### 3.4 Interpretability analysis in the model

**Figure 4:**
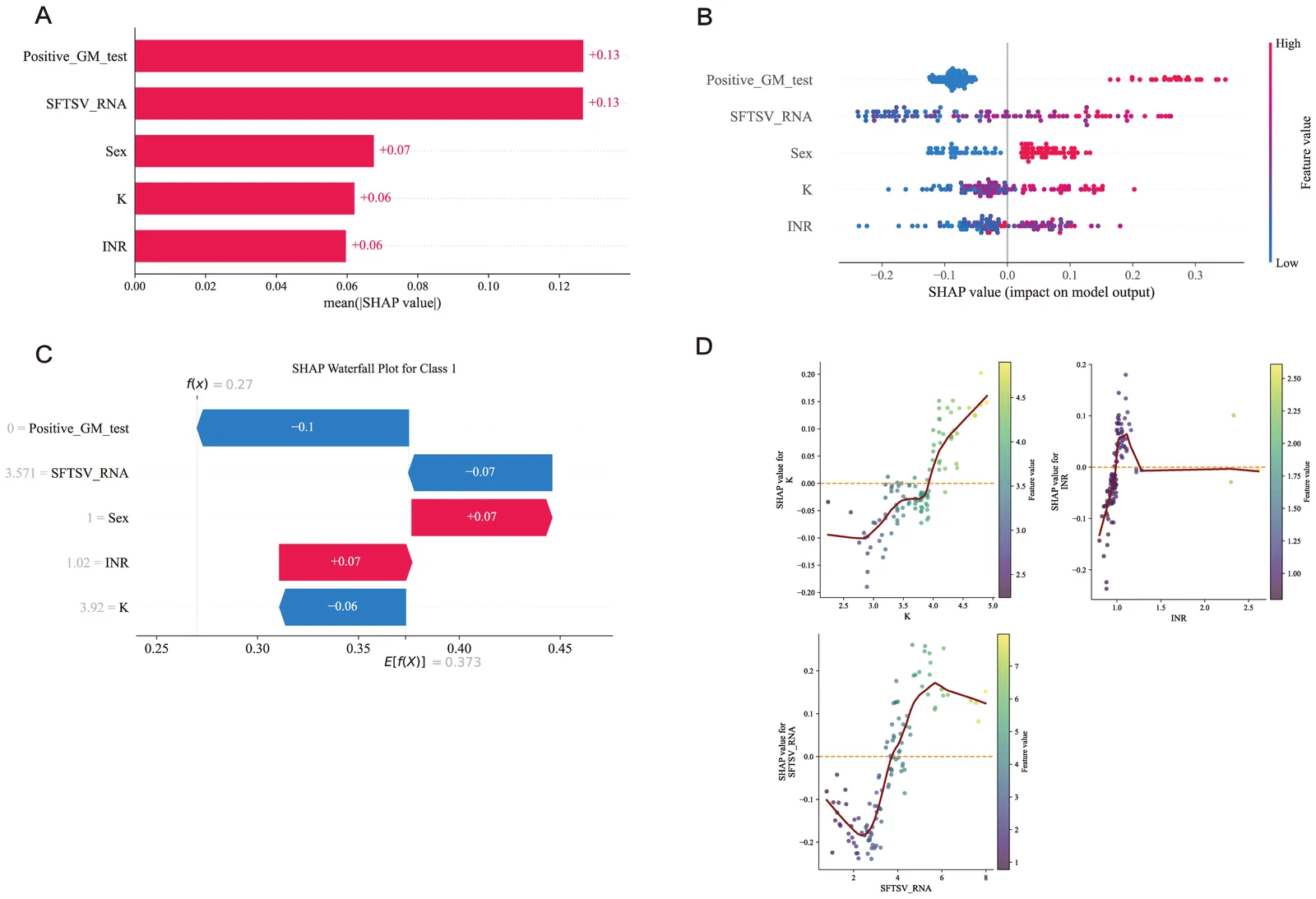
SHAP interpretability analysis of feature contributions for the Random Forest model. **(A)** SHAP feature-importance bar plot; **(B)** SHAP beeswarm plot; **(C)** SHAP waterfall plots for representative Class 1; **(D)** SHAP dependence plots.

The global feature importance ranking demonstrated GM test positivity and SFTSV RNA load as the most influential predictors contributing to the model’s output for IPA (**Figure 4A**).

The SHAP beeswarm plot further demonstrated both directionality and inter-individual heterogeneity by visualizing the impact of each feature on the model’s output (**Figure 4B**). In this plot, the color represented the feature value, with red indicating a high value and blue indicating a low value. A positive Shapley value for a feature indicated an increased risk of IPA, whereas a negative value suggested a decreased risk. The SHAP waterfall plot visualized the direction and degree of the influence of different features of the 1st participant in the study population on the final predicted value (**Figure 4C**). The SHAP dependence plots (**Figure 4D**) illustrated the nonlinear relationships between continuous predictors and IPA risk, providing detailed insights into how individual features influenced model outputs across their value ranges. Specifically, this figure revealed that elevated levels of serum potassium and SFTSV RNA load, along with INR values ranging from 1.0 to 1.4, were associated with a heightened risk of IPA in patients with SFTS.

### 3.5 Sensitivity analysis

**Table 4** presented the results of the sensitivity analysis after excluding GM test positivity from all models. Using the same model-development framework, the reconstructed models showed attenuated discrimination compared with the primary analysis, with AUCs ranging from 0.700 to 0.775. These findings indicate that the models retained moderate discriminatory ability after exclusion of GM test positivity, suggesting that predictive performance was supported by multiple clinical variables rather than driven solely by GM test positivity.

**Table 4:** Sensitivity analysis.

Algorithm	Primary AUC (incl. GM test positivity)	Sensitivity AUC (excl. GM test positivity)	Change in AUC
Logistic	0.824	0.775	-0.049
Decision Tree	0.857	0.700	-0.157
Random Forest	0.871	0.733	-0.138
XGBoost	0.862	0.770	-0.092
LightGBM	0.836	0.717	-0.119
SVM	0.838	0.749	-0.089
ANN	0.826	0.748	-0.078

Comparison of AUC values between primary analysis (including GM test positivity) and sensitivity analysis (excluding GM test positivity). Changes in AUC reflect the influence of GM test positivity on model discrimination.

## 4 Discussion

In this retrospective study, we developed and internally evaluated seven ML models to establish a clinically interpretable model for the early identification of IPA in SFTS patients. By incorporating accessible features collected at least 72 hours prior to the diagnosis of IPA, our model has the potential to function as an adjunctive early-warning tool that complements existing diagnostic criteria and may assist in the early identification of high-risk patients.

In recent years, studies on the screening of risk factors for SFTS-associated IPA have remained limited, and only one predictive model has been reported. Yan et al. developed a nomogram based on four predictors, including maximum viral load, WBC count, BUN, and APTT, and achieved a validation AUC of 0.75 (Yan et al., 2025). Notably, both studies adopted similar diagnostic criteria and included cohorts of comparable size, enhancing the comparability of the findings. However, the predictors retained in the final model differed between studies, likely reflecting differences in patient characteristics and variable selection strategies. Furthermore, differences in patient sources and baseline disease severity may also have influenced model performance. Therefore, these performance metrics should be interpreted and compared with caution. Future external validation across diverse clinical settings is warranted to further evaluate the generalizability and clinical applicability of the model.

While several studies have suggested that males are more susceptible to IPA (Shi et al., 2022; Teng et al., 2025), our findings indicate a higher vulnerability in female patients. Female sex was retained by the model despite the lack of a significant between-group difference at baseline. This finding may reflect interactions with other predictors rather than an independent association and should be interpreted cautiously.

Serum potassium levels and INR may indirectly reflect disease severity in patients with SFTS. Given that critically ill patients with SFTS are more susceptible to developing IPA, these variables may contribute to the prediction of IPA risk (Wang et al., 2024). Previous large-scale studies have demonstrated a U-shaped relationship between serum potassium levels and mortality, with both hypokalemia and hyperkalemia associated with a poor prognosis (Noori et al., 2022). In the context of SFTS, Zheng et al. incorporated serum potassium into a Boosted Topology Model for early prediction of life-threatening conditions, suggesting its potential prognostic relevance, although the relationship between serum potassium levels and mortality risk was not specifically examined (Zheng et al., 2023). In our analysis, higher serum potassium was associated with an increased risk of IPA. This finding may reflect the impact of impaired renal potassium excretion and electrolyte dysregulation (Wang et al., 2025). Nevertheless, the biological mechanisms linking elevated serum potassium levels to adverse outcomes in SFTS have not been fully elucidated and require further investigation. Beyond electrolyte disturbances, prolonged INR indicates the development of disseminated intravascular coagulation and endothelial damage induced by severe systemic inflammatory responses, both of which are closely associated with the severity of SFTS (Xia et al., 2024; Zhang et al., 2025).

Accumulating evidence has identified SFTSV viral load as a critical determinant of disease progression and clinical prognosis in infected patients (Chen et al., 2025). Recent studies have further demonstrated that SFTSV nonstructural protein (NSs) sequesters TANK-binding kinase 1 (TBK1), abrogating its inhibitory effect on NF-κB signaling and thus driving the uncontrolled release of pro-inflammatory mediators, such as IL-6, TNF-α, and MCP-1 (Khalil et al., 2021; Guo and Jiang, 2025). This systemic pro-inflammatory surge triggers vascular compromise (Westover et al., 2019) and induces profound transcriptional remodeling in low-density neutrophils (LDNs), with the concurrent upregulation of inflammatory and type I interferon response gene sets (Zhao et al., 2025a). Such profound immune dysregulation ultimately impairs host defense mechanisms, thereby elevating the risk of secondary opportunistic infections, including IPA.

This study has several limitations. First, the retrospective single-center design may have introduced selection bias and may limit the generalizability of the findings. Although internal validation and bootstrap resampling were performed, the model has not yet undergone external validation in independent cohorts. Second, GM positivity was retained as a predictor despite being part of the diagnostic criteria of probable IPA, which may have introduced a degree of incorporation bias. Although sensitivity analysis excluding GM demonstrated acceptable model performance, this finding should therefore be interpreted with caution. Third, all predictors were measured only once at baseline, and dynamic changes during hospitalization were not captured. As a result, the model cannot be used as a real-time monitoring tool and may not fully reflect changes in patient status over time. Given these limitations, our current model should be regarded as a preliminary, complementary tool for early diagnostic evaluation and risk stratification rather than a substitute for comprehensive clinical judgment of the attending physician. Future multicenter studies involving larger cohorts from diverse geographic regions and healthcare settings are warranted to externally validate the model and further evaluate its generalizability.

## 5 Conclusion

In conclusion, we developed and internally validated an interpretable RF model for the early diagnosis of IPA in patients with SFTS. By integrating five key predictors—sex, serum potassium, INR, GM test positivity, and SFTSV RNA load—the model demonstrated promising predictive performance in our cohort. This may assist in the early identification of patients at increased risk of IPA and complement existing diagnostic approaches. Future prospective studies are warranted to externally validate this model in larger, independent SFTS cohorts.

## Data Availability

The raw data supporting the conclusions of this article are available from the corresponding author upon reasonable request.
