# Correction: Development and validation of an interpretable machine learning model for the early diagnosis of invasive pulmonary aspergillosis in patients with severe fever with thrombocytopenia syndrome: a retrospective cohort study

**DOI:** 10.3389/fcimb.2026.2027760

**Published:** 2026-10-26

**Authors:** Shuwen Ding, Ruize Ma, Wan Peng, Yanli Xu, Ling Lin, Zhihai Chen

**Affiliations:** 1 National Key Laboratory of Intelligent Tracking and Forecasting for Infectious Diseases, Beijing Ditan Hospital, Capital Medical University, Beijing, China; 2 Department of Infectious Diseases, Yantai City Hospital for Infectious Disease, Yantai, China

**Keywords:** early diagnosis, invasive pulmonary aspergillosis, machine learning, predictive models, severe fever with thrombocytopenia syndrome

In the published article, an incomplete **Funding** statement was provided. The correct **Funding** statement reads: The author(s) declared that financial support was received for this work and/or its publication. This work was supported by the National Science and Technology Major Project of China (Grant No. 2018ZX09711003); the National Natural Science Foundation of China (Grant No. 82072295); and the National Key Research and Development Program of China (Grant No. 2022YFF1203201).

The original version of this article has been updated.

